# Editorial: Advances toward improved understanding and treatment of uncommon ovarian cancer types and subtypes

**DOI:** 10.3389/fonc.2024.1519252

**Published:** 2024-12-09

**Authors:** Robert L. Hollis, Mignon D. J. M. van Gent

**Affiliations:** ^1^ The Nicola Murray Centre for Ovarian Cancer Research, Cancer Research UK Scotland Centre, Institute of Genetics and Cancer, University of Edinburgh, Edinburgh, United Kingdom; ^2^ Department of Gynaecologic Oncology, Center for Gynaecologic Oncology Amsterdam, Cancer Center Amsterdam, Amsterdam University Medical Centres, Amsterdam, Netherlands

**Keywords:** ovarian cancer, uncommon tumors, endometrioid ovarian carcinoma, low grade serous ovarian carcinoma, clear cell ovarian carcinoma (CCOC), ovarian carcinosarcomas (OCS), ovarian sex cord-stromal tumor

## Introduction

Ovarian cancer is an umbrella term for a multitude of distinct disease entities identified in and around the ovary, fallopian tube and peritoneum. These include epithelial ovarian cancers (ovarian carcinomas), of which there are six major types: high grade serous (HGSOC), endometrioid (EnOC), clear cell (CCOC), mucinous (MOC), low grade serous (LGSOC) and ovarian carcinosarcoma (OCS) (1). Non-epithelial cancers include malignant germ cell tumors (teratoma, dysgerminoma, yolk sac tumor and others), sex chord stromal tumors (granulosa cell tumors, Sertoli-Leydig cell tumors and more), Brenner tumors and mesenchymal tumors, among others (2). These various types have been shown to arise from distinct developmental origins, have unique molecular profiles, varied response rates to conventional and targeted therapies, and distinct overall clinical behavior (2–8).

HGSOC is by far the commonest, and the vast majority of research has accordingly focused on this tumor type. These studies have advanced our knowledge of HGSOC at the genomic, transcriptomic and proteomic levels (4, 9–11), identifying therapeutically–exploitable disease biology that has led directly to the design and utilization of additional targeted treatment strategies, including poly(ADP–ribose) polymerase (PARP) inhibitors (12, 13). After approximately 30 years of limited progress in improving ovarian cancer survival, the integration of these agents into routine clinical practice is now shifting the survivorship landscape in HGSOC.

However, progress within the other, less common, ovarian cancer types has been lacking, and many remain critically understudied with a corresponding lack of targeted therapeutic options. Indeed, the fundamental molecular landscape in many of these tumor types have either only recently been established, or have yet to be described in large numbers of samples (3, 7, 8, 14, 15). In this Research Topic, we aimed to provide a platform for communication of research in uncommon and understudied forms of ovarian cancer, in the hope of advancing our understanding of these discrete disease entities.

## Ovarian carcinosarcoma

OCS represents approximately 4% of ovarian cancer diagnoses, is characterized by the presence of both high grade carcinomatous and high grade sarcomatous components (2), and is exceptionally aggressive (median survival <2 years) with higher levels of intrinsic chemoresistance compared to HGSOC (16).

Three contributions on OCS are presented in this Research Topic that augment our current understanding of this uncommon and aggressive tumor type. Zheng et al. report a case of a 76 year–old female diagnosed with FIGO stage IIIC OCS. The report provides an excellent example of OCS histopathology, with contrasting cytokeratin immunohistochemical profiles between carcinomatous (CK+) and sarcomatous components (CK–), but shared aberrant p53 immunophenotype indicative of *TP53* mutation. They also demonstrate the presence of chondrosarcomatous differentiation, which has been reported as the most frequent heterologous element in OCS (16).

In a clinical cohort study, McFarlane et al. make use of two contrasting data sources to compare the clinical behavior of OCS patients versus those with other ovarian carcinomas: one from The Edinburgh Ovarian Cancer Database, the other from Surveillance Epidemiology and End Results (SEER) database. The findings identify OCS as the histotype with the least favorable overall survival profile, and this is especially the case in the context of early stage diagnosis, with FIGO stage I–II OCS patients demonstrating a median survival time of just two years in their primary cohort. The study also demonstrates that OCS patients represent an older patient population compared to other histotypes, with the median age at diagnosis being 67 years.

Finally, a molecular profiling study is presented by Dhillon et al., analyzing a cohort of OCS samples by targeted sequencing and immunohistochemical profiling. They show that the *TP53* mutation rate in this tumor type is high, but that a minority of cases (15–20%) are p53 wildtype. The p53 wildtype population demonstrated poorer survival, and this is one of the first reported molecular prognostic factors in OCS. Moreover, they demonstrate that a proportion of OCS harbor *BRCA1*/*2* mutation, highlighting the potential for some OCS patients to benefit from PARP inhibition. The *BRCA1*/*2*–mutant cases were suggested to experience more favorable survival, with 100% 3–year survival, though the number of *BRCA1*/*2*–mutant cases was limited.

## Low grade serous ovarian carcinoma

LGSOC accounts for 3–5% of ovarian cancer diagnoses, demonstrates high levels of intrinsic chemoresistance, and affects younger women compared to HGSOC. Advancements have recently been made in treatment of LGSOC, with MEK inhibitors now recognized as a useful therapeutic option at recurrence (17), and endocrine maintenance therapy demonstrating substantial clinical activity (18).

A case report by Al–Aloosi et al. depicts an ex vivo drug testing study performed on organoids derived from a metastatic site of a patient with progressing LGSOC. Molecular tumor testing had previously revealed a somatic Y537S *ESR1* mutation likely associated with acquired resistance to letrozole, alongside absence of *KRAS*, *BRAF* or *NRAS* mutation. Characterization of organoid sensitivity to a panel of compounds and rational combinations resulted in the subsequent use of the endocrine therapy fulvestrant with the mTOR inhibitor everolimus. The authors report CA125 stabilization and a disease control period of 7 months on this treatment.

A sub–cohort analysis of a phase I study, presented by Nakamura et al., examines the safety of cisplatin–doxorubicin pressurized intraperitoneal aerosolized chemotherapy (PIPAC) in four heavily pre–treated LGSOC patients. The authors report the regimen to be well tolerated, and recommend further consideration of this strategy for recurrent LGSOC, where new treatment options are urgently needed to improve patient outcomes.

## Endometriosis–associated ovarian cancers: endometrioid and clear cell carcinoma

EnOC and CCOC each represent up to 10% of ovarian cancer diagnoses, and are both recognized to be related to endometriosis. CCOC is highly chemoresistant, while EnOC reportedly demonstrates intermediate chemosensitivity that is lower than that of HGSOC. Both EnOC and CCOC are usually diagnosed at earlier stage compared to HGSOC (1), and both are among the epithelial types that appear to benefit most from complete surgical resection (19).

Two cohort studies using data from the SEER database are presented by (Liu et al. and Tian et al.). The former constructs a prognostic nomogram for CCOC showing the importance of log odds of positive lymph nodes (LODDS) in predicting ovarian cancer–specific survival, the latter uses a cohort of 4257 CCOC patients to demonstrate improvement in survival across time within the diagnosis period of 2000–2015.

Two review articles cover key topics in the field of endometriosis–associated ovarian cancers (Chen et al., Tang and Bian). Both cover key research progress made within CCOC and EnOC. In particular, they cover our contemporary understanding of the molecular drivers in these tumor types, key risk factors and summarize progress in the diagnosis and management of CCOC and EnOC.

Finally, a case report from Zhao et al. presents an individual with simultaneous EnOC and CCOC alongside endometriosis. Complementary molecular analysis demonstrated shared *ARID1A*, *KRAS*, *PIK3CA* and other mutational events in the two malignant populations, evidencing their clonal relationship.

## Non–epithelial tumors

There are a large number of non–epithelial tumor types diagnosed at the ovary, the majority of which are poorly characterized at the molecular level. Many of these types are rare individually, but collectively non–epithelial tumors account for 10% of ovarian cancer cases. Accordingly, approximately 30,000 new diagnoses of these cancers are made worldwide each year (20). The vast majority of research beyond HGSOC has focused on other epithelial cancer types, leaving non-epithelial tumors critically understudied.

A review of ovarian steroid cell tumors, presented by Wei and Fadare, explores the clinical, radiological and histopathological features of these tumors alongside an overview of known molecular features. A retrospective study presented by Marino et al. examines patients with stage I immature teratoma that underwent either adjuvant chemotherapy or surveillance following fertility–sparing surgery, demonstrating excellent outcomes in both groups across the study period (100% overall survival in both groups, 87% and 90% disease–free survival in the surveillance and chemotherapy–treated groups, median follow–up time >15 years).

Two case reports of uncommon phenomena occurring in patients subsequent to teratoma diagnoses are presented: Tao et al. report a case of growing teratoma syndrome following treatment for immature teratoma with a review of the literature, highlighting this rare phenomenon, of which there is currently limited awareness. A second case report presents an individual with ovarian yolk sac tumor subsequent to mature cystic teratoma (Li et al.). Both of these clinical situations are uncommon, but worthy of highlighting to clinicians.

## Variants of HGSOC

While HGSOC has received substantial research attention to date, subtypes within HGSOC are now widely recognized at the molecular level. In particular, around 50% of HGSOC are homologous recombination DNA repair deficient (HRD) and these tumors have been the focus of intense study (1). These investigations have culminated in the discovery and integration of PARP inhibitors into ovarian cancer management, which are most efficacious in HGSOC patients with identifiable HRD (13, 21). By contrast, the various homologous recombination repair proficient (HRP) molecular subtypes have been less extensively studied, such as those that demonstrate copy number gain of *CCNE1*.

Stiegeler et al. provide a comprehensive overview of HRP HGSOC in their review article, highlighting key potential therapeutic strategies particularly in the context of platinum–resistant relapse. The authors include targeted inhibitors of CDK1/2, WEE1, PI3K, AKT and ATR as options in their potential future HRP–HGSOC treatment algorithm, alongside the folate receptor alpha–targeted antibody–drug–conjugate Mirvetuximab.

A case report from Giancontieri et al. depicts an unusual case of high grade serous carcinoma of unknown primary at an inguinal node. Pathological examination demonstrated WT1, CK7 and PAX8 positivity, leading to a suspicion of tubo–ovarian origin. Subsequent surgery revealed only a serous tubal intraepithelial carcinoma (STIC), and a multidisciplinary team determined occult non–invasive STIC with node metastasis, and the authors propose this is likely from exfoliation and peritoneal spread rather than lymphatic spread.

An *in vitro* study presented by Iida et al. describes suppression of CRY1 as a potential mechanism by which anti–angiogenics may improve the efficacy of PARP inhibition in HRP HGSOC. They propose that CRY1 inhibition may be a potential strategy for improving PARP inhibitor efficacy, particularly for tumors that are considered HRP.

## Concluding remarks

Uncommon forms of ovarian cancer are critically understudied, despite collectively representing around one third of ovarian cancer diagnoses, and some of these patient groups are markedly underserved by currently available treatment regimens. If patients diagnosed with these tumor types are to benefit from expanded treatment options in a similar manner to those with more common HGSOC, then it is clear that additional research attention will be critical for defining targetable disease drivers.
